# Study of hospitalization and mortality in Korean diabetic patients using the diabetes complications severity index

**DOI:** 10.1186/s12902-020-00605-5

**Published:** 2020-08-10

**Authors:** Hyunju Yoo, Eunjung Choo, Sukhyang Lee

**Affiliations:** Division of Clinical Pharmacy, College of Pharmacy, Ajou University, 206 Worldcup-ro Yeongtong-gu, Suwon, 16499 South Korea

**Keywords:** Diabetes mellitus, National Health Insurance Database, Diabetes complications, Korea

## Abstract

**Background:**

The prevalence of type 2 diabetes mellitus (T2DM) is expected to increase from 7.7% in 2017 to 8.4% in 2045 worldwide. Diabetes complications contribute to morbidity and mortality. To evaluate whether the diabetes complications severity index (DCSI) was associated with increased risks of mortality and hospitalization.

**Methods:**

A retrospective cohort study was conducted using the National Health Insurance Database (NHID) sample cohort of 1,102,047 patients (2002–2015) in Korea. Diabetes complications were evaluated at 2 years after the initial diagnosis and during the subsequent follow-up period (mean duration 6.56 ± 2.81 years). The type and severity of complications were evaluated on the basis of the International Classification of Disease Ninth (ICD-9) codes used in DCSI with 7 categories and 55 subcategories of complications. The Cox proportional hazard and Poisson regression models were used to evaluate the mortality and hospitalization rates. The incidence and relative risk of diabetes complications as well as the risk of mortality and hospitalization were the main outcome measures.

**Results:**

A total of 27,871 patients were finally included and grouped by the number of complications present at 2 years. Four hundred ninety patients (5.37%) died without complications, 659 (7.31%) died with one complication and 1153 (11.85%) died with two or more complications. As DCSI at index date increased, the risk of additional new diabetes complications increased by 26% [relative risk (RR) 1.26, 95% CI 1.25–1.27]. The risks of mortality and hospitalization were linearly related to DCSI [hazard ratio 1.13 (95% CI 1.11–1.16), relative risk 1.04 (95% CI 1.03–1.06)].

**Conclusions:**

Patients with higher incidence and severity of diabetes complications have increased risks of mortality and hospitalization.

## Background

The prevalence of type 2 diabetes mellitus (T2DM) is expected to rise from 7.7% in 2017 to 8.4% in 2045 worldwide. The number of deaths due to diabetes was estimated to be 5 million, accounting for 9.9% of the total number of deaths worldwide in 2017 [1, 2].

Diabetic complications could be identified at the time of DM diagnosis with pathophysiological changes ever since the stage of prediabetes. Di Pino et al. reported that the glycated hemoglobin A_1C_ (HbA_1C_) and receptors for advanced glycation end products (RAGEs) were related to cardiovascular disease in subjects with prediabetes based on the impaired fasting glucose or glucose tolerance test. Previous studies showed the association of the glycation and inflammation with morbidity and mortality in T2DM [3–6]. The goal of diabetes treatment is not only to maintain an optimal blood glucose level but also to prevent DM complications, such as nephropathy, neuropathy, retinopathy, and cardiocerebrovascular disease [7–9]. The 10-year risk of cardiovascular disease (CVD 10-year risk) was used as an index to evaluate the risk of developing CVD, which included age, male, DM, hypertension, and dyslipidemia as risk factors [10–12]. T2DM is an independent risk factor that increases the risk of CVD by approximately 2 fold and CVDs are the leading cause of deaths in diabetic patients [13]. Diabetic nephropathy is the most common complication that occurs in 20–40% of diabetic patients and progresses to end-stage renal failure requiring dialysis or kidney transplantation. In the case of neuropathy, symptoms are very diverse, and blood glucose control can delay but not prevent nerve damage. Diabetic retinopathy is a common cause of blindness and is closely related to the duration of diabetes and failure to control blood glucose levels [9]. The medical cost, mortality, and disease burden of DM increase as the number of complications increases [14–16].

A study using data from patients with diabetes at nine primary care clinics reported the diabetes complications severity index (DCSI), which reflects the severity of the complications, and identified an association of the frequency and severity of diabetes complications with the risks of mortality and hospitalization; DCSI was demonstrated to be a good indicator of the association between diabetes complications and mortality [14]. Rosenzweig et al. reported the use of DCSI for evaluation of healthcare costs and the management of comorbidities in patients with diabetes. The medical costs were 10 times higher for diabetic patients in the very high-risk group than in the low-risk group [17]. Several studies have evaluated the risk of diabetes complications using the real world data of diabetic patients. Selby et al. developed a prediction rule to identify high-risk factors associated with diabetes complications using the large clinical database of Kaiser Permanente. They found that a history of complications was the strongest risk factor for the incidence of diabetes complications [18]. Clinical significance of DCSI needs to be assessed in usefulness for evaluating the risks of mortality and hospitalization using the real world big data in a Korean population.

## Methods

### Aim of the study

This retrospective cohort study aimed to investigate whether diabetes complications severity index (DCSI) is associated with all-cause mortality and hospitalization rates in patients with T2DM in a Korean population. DCSI was based on the frequency and pattern of complications in diabetic patients at 2 years after T2DM diagnosis. Also, we analyzed factors that may affect newly developed complications beyond 2 years from the initial diagnosis.

### Design and setting of the study

A retrospective cohort study was conducted using the Korean National Health Insurance Service–National Sample Cohort (NHIS–NSC) 2.0 which was a population-based database with 1,102,047 patients (2002–2015). The NHIS cohort 2.0 comprises the insurance claim database of approximately 1 million individuals, accounting for 2% of the Korean population who retained the status of health insurance members and medical entitlement. This sample cohort was selected by stratified random sampling according to sex, age, eligibility status, and income levels to represent the Korean population that comprised nearly 50 million individuals. They were followed up for 14 years unless an individual’s eligibility was disqualified due to death or emigration. The insurance claim database comprised information on demographic and clinical characteristics and medical record data, including age; sex; dates of birth and death; clinic, hospital, and pharmacy visit information; disease diagnosis; medical care procedures; and prescribed medications. The database comprises five tables that provide information on the qualification of insurance, birth and death, medical care, health examination, medical institutions, and duration of hospitalization. The death of the subject is linked to the cause of death at the National Statistical Office to identify the date and cause of death. Before the study, individual information in the NHIS–NSC database was de-identified to protect the patients’ privacy, and patients in the database only had encrypted identification numbers [19, 20].

**Fig. 1:**
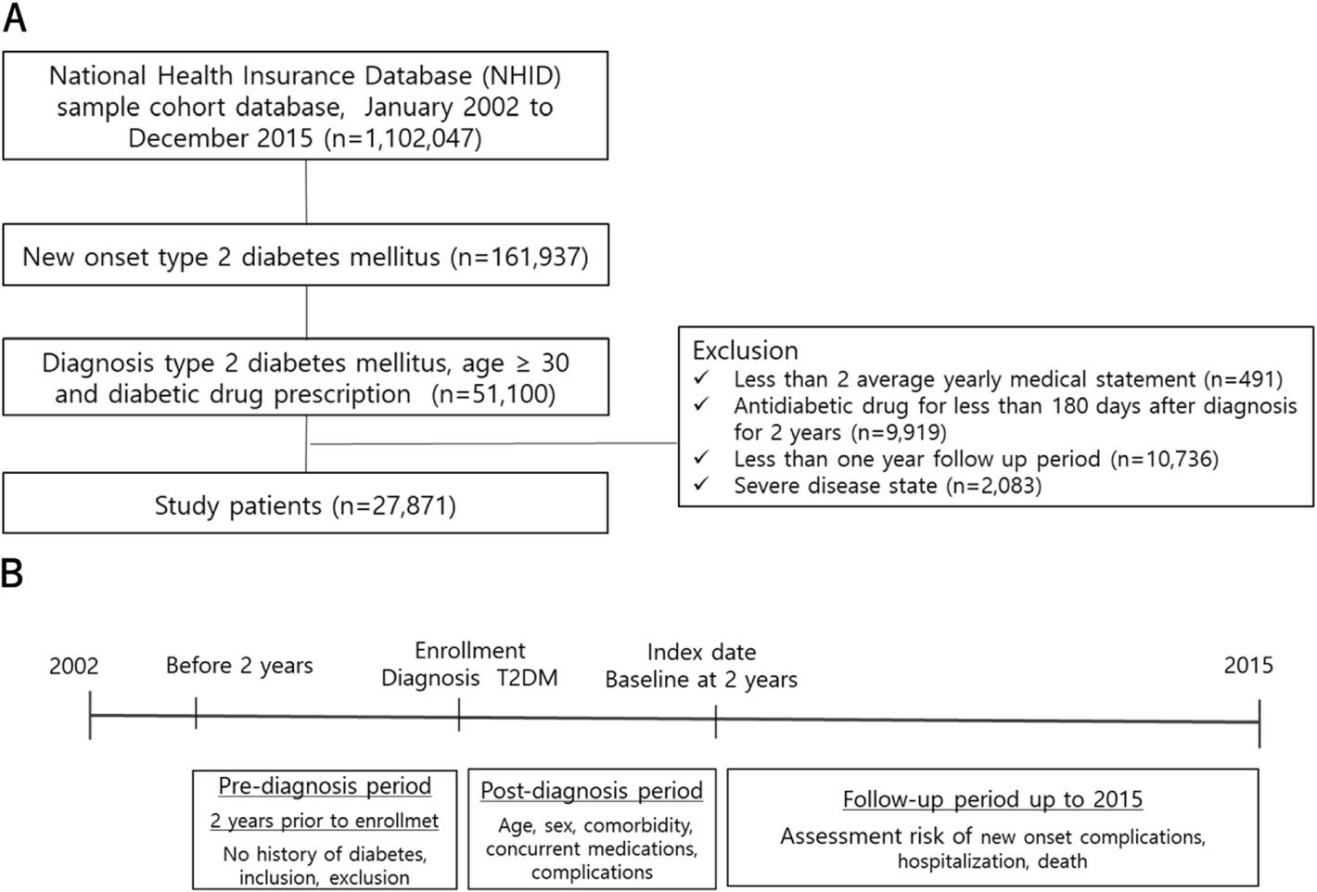
Flow chart of the study population (**a**) and schematic description of the study design (**b**)

The enrollment date was defined as the date of diabetic diagnosis and diabetes medication. The index date was defined as 2 years after the diagnosis of diabetes. The prediagnosis and postdiagnosis period were 2 years before and after the diagnosis of diabetes, respectively. The follow-up period, from the index date to 2015, was used to assess the complications, deaths, and hospitalizations that occurred during this period (Fig. 1).

Data regarding demographic characteristics, comorbidities, concomitant medications, and clinical procedures were collected. Comorbidities before the index date were identified, and the Charlson comorbidity index (CCI) score was calculated for the postdiagnosis period [21]. The concomitant medications used in the postdiagnosis period were aspirin, cilostazol, sarpogrelate, angiotensin-1 converting enzyme inhibitor or angiotensin-2 receptor blocker (ACEI/ARBs), dihydropyridine calcium channel blocker (DHP-CCB), and statin. Antidiabetic drug adherence was evaluated in patients with a medication possession rate (MPR), which was defined as the ratio (A/B) of the total prescription days (A) to the duration from the first to the last date of drug use during the postdiagnosis period (B).

### Inclusion and exclusion criteria

The inclusion criteria in this study were patients aged ≥30 years who were diagnosed with T2DM (E11.0) and claim data for prescriptions of antidiabetic medication for 180 days or longer. The initial diagnosis of diabetes was defined as newly diagnosed patients for 2 years before enrollment in the study. Patients included when they had a diagnosis of diabetes mellitus, prescription of diabetes medications, and medical insurance claim records more than twice a year.

The exclusion criteria were patients with chronic kidney disease stage 5 (ICD-10 codes, N18.5), extracorporeal dialysis (ICD-10 codes, Z49.1), other dialysis (ICD-10 codes, Z49.2), kidney transplant status (ICD-10 Z94.0), liver dysfunction (ICD-10 codes, K72.1), and cancer (ICD-10 codes, C00-C80). Patients with end-stage diseases were excluded to reduce bias from the incidence of complications, hospitalization, and deaths due to underlying diseases other than diabetes. Patients were excluded if they took antidiabetic drugs for less than180 days and died before the index date during the 2-year postdiagnosis period.

### Outcomes

The type and frequency of diabetes complications were analyzed at the index date of 2 years after T2DM diagnosis (baseline) and during the follow-up period. Diabetes complications were grouped into 7 categories (nephropathy, neuropathy, retinopathy, cerebrovascular disease, cardiovascular disease, peripheral vascular disorder, and metabolic diseases) and 55 subcategories. The trends of changes in the complications were evaluated from the index date to the end of the follow-up period. Complications were also analyzed using DCSI that reflected the influence of diabetes complications. DCSI was based on a scale ranging from 0 to 2 for each complication abnormality, with a total maximum score of 13 (Supplement Table 1).

New onset complications were defined as the appearance of new diagnostic codes for complications in the DCSI category or subcategories during subsequent periods. These were new diagnostic codes for complications that did not occur before the index date. The pattern of drug use was evaluated in patients with or without new-onset complications. The use of medication for ≥90 days was evaluated for 1 year before the index date. The occurrence of complications was evaluated to identify its association with age, sex, comorbidity, the type of antidiabetics [metformin, sulfonylurea (SU), dipeptidyl peptidase-4 inhibitor (DPP4I), α-glucosidase inhibitor, thiazolidinedione, and insulin], the pattern of pharmacotherapy (monotherapy or dual combination therapy), and concomitant medications (Supplement Table 2).

The mortality and the frequency and duration of hospitalization due to all causes were analyzed in patients with none, one, or two or more complications during the follow-up period. The association of hospitalization and mortality with age, sex, insulin use, number of complications, and DCSI was evaluated during the follow-up period. The survival analysis of the patients was performed on the basis of the number of complications at baseline during the follow-up period. Receiver operating characteristics analysis was performed to determine the usefulness of DCSI in evaluating the association of diabetes complications with mortality.

### Statistical analyses

The baseline characteristics were expressed as mean ± standard deviation (or median and interquartile range) for continuous variables and as the frequency with percentages for categorical variables, as applicable. The continuous variables were compared using Student’s t-test or one-way analysis of variance, and the categorical variables were compared using the chi-squared test. Poisson regression models were used to evaluate the new-onset diabetes complications and hospitalizations. The Kaplan–Meier method and log-rank test were used to evaluate the mortality according to the number of diabetes complications, and the Cox proportional hazard model was used to predict mortality. The risk of hospitalization or mortality in diabetic patients was analyzed by adjusting the covariates to account of effect age, sex, and insulin use.

## Results

### Characteristics of the study population

A total of 161,937 patients with new diagnosis T2DM at enrollment were selected from the NHIS database. Among them, 51,100 patients were identified on the basis of inclusion criteria aged ≥30 years with a prescription for antidiabetic medications. Further, 23,229 patients were excluded on the basis of the following exclusion criteria: less than 2 insurance claims each year (*n* = 491 patients), took antidiabetic drugs for less than180 days (*n* = 9919 patients), and the presence of an end-stage disease (*n* = 2083 patients) (Fig. 1).

The final study population comprised 27,871 patients; their mean age was 57.28 years, 54.29% men and 45.71% women, and the mean duration of follow up was 6.56 ± 2.81 years. The major concomitant diseases were hypertension in 66.94% and dyslipidemia in 72.57% of patients. During the 2-year postdiagnosis period, 70.06% of the patients had taken metformin, 53.62% SU, 15.48% DPP4I, and 2.57% insulin. The adherence of antidiabetic drugs was 77.86% measured as medication possession rate. The concomitant medication was ACEI/ARB in 47.81% of the patients, DHP-CCB in 40.67%, and statin in 46.52%. Of 27,871 patients, 9130 patients (32.76%) had no complication, 9015 patients (32.35%) had one, and 9726 (34.90%) patients had two or more complications at the index date of 2 years postdiagnosis. Older patients were found to have more complications than younger patients. The CCI score for coexisting diseases was 1.66 ± 1.00 in patients with no complication, 2.62 ± 1.35 in patients with one, and 4.12 ± 1.82 in patients with two or more complications (Table 1).

**Table 1:** Baseline characteristics at 2 years after T2DM diagnosis

Characteristics	All subjects (27,871)	Number of complications		
		None (9130)	1 (9015)	≥ 2 (9726)
Age (year)				
Mean ± SD	57.28 ± 11.97	54.30 ± 11.42	57.05 ± 11.87	60.30 ± 11.84
Median, IQR	57, 48–66	53, 46–62	56, 48–66	61, 52–69
< 65	19,643 (70.48)	7327 (80.25)	6448 (71.53)	5868 (60.33)
≥ 65	8228 (29.52)	1803 (19.75)	2567 (29.47)	3858 (39.67)
Gender, n (%)				
Men	15,132 (54.29)	5526 (60.53)	4930 (54.69)	4676 (48.08)
Women	12,639 (45.71)	3604 (39.47)	4085 (45.31)	5050 (51.92)
CCI, mean ± SD	2.83 ± 1.77	1.66 ± 1.00	2.62 ± 1.35	4.12 ± 1.82
Follow up period, year ±SD	6.56 ± 2.81	6.61 ± 2.86	6.56 ± 2.80	6.52 ± 2.77
Median, IQR	6.3, 4.1–9.0	6.3, 4.1–9.2	6.3, 4.2–7.0	6.3, 4.1–8.9
DCSI, n (%)	1.44 ± 1.46	0	1.14 ± 0.36	3.05 ± 1.19
Comorbidity, n (%)				
Hypertension	18,657 (66.94)	5164 (56.56)	5914 (65.60)	7579 (77.93)
Dyslipidemia	20,225 (72.57)	5760 (63.09)	6529 (72.42)	7936 (81.60)
Stroke	2485 (8.92)	–	423 (4.69)	2062 (21.20)
Myocardial infarction	566 (2.03)	–	172 (1.91)	394 (4.05)
Antidiabetic drug adherence				
Mean ± SD,	77.86 ± 28.14	76.26 ± 28.54	77.86 ± 27.99	79.36 ± 27.81
Median, IQR	90.41, 66.84–90.41	90.13, 65.75–98.63	98.63, 66.02–90.41	90.41, 71.50–98.63
Medications, n (%)				
Antidiabetics				
Metformin	19,526 (70.06)	6418 (70.30)	6403 (71.03)	6705 (68.94)
Sulfonylurea	14,945 (53.62)	4989 (54.64)	4774 (52.96)	5182 (53.28)
DPP4-I	4314 (15.48)	1365 (14.95)	1439 (15.96)	1965 (20.20)
Thiazolidinedione	1703 (6.11)	483 (5.29)	531 (5.89)	689 (7.08)
Insulin	715 (2.57)	113 (1.24)	156 (1.73)	446 (4.59)
Cardiovascular agents				
Aspirin	8791 (31.54)	1761 (19.29)	2803 (31.09)	4227 (43.46)
Clopidogrel	1754 (6.29)	31 (0.34)	462 (5.12)	1261 (12.97)
Cilostazol	1068 (3.83)	72 (0.79)	244 (2.71)	752 (7.73)
Sarpogrelate	726 (2.60)	58 (0.64)	167 (1.85)	501 (5.15)
ACEI/ARB	13,325 (47.81)	3534 (38.71)	4212 (46.72)	5579 (57.36)
DHPCCB	11,336 (40.67)	3270 (35.82)	3594 (39.87)	4472 (45.98)
Statin	12,966 (46.52)	3556 (38.95)	4081 (45.27)	5329 (54.79)

Study subject’s characters were not normal distribution (Normality test - Kolmogorov-Smirnov)

Per oral drug use history is prescribed for more than 90 days in the previous year at 2 years after diagnosis T2DM

*Abbreviation*: *CCI* Charlson comorbidity index, *DCSI* diabetes complication severity index, *DPP4-I* dipeptidyl peptidase-4 inhibitor, *ACEI/ARB* angiotensin-1 converting enzyme Inhibitor/angiotensin-2 receptor blocker, *DHPCCB* dihydropyridine-calcium channel blocker

### Total diabetes complications at 2 years postdiagnosis and during the follow-up period

The number of patients with complications at the index date the 2-year postdiagnosis period was total 18,741. Patients with no complication were 9130 (32.76%) at index date and 3714 (13.33%) during the follow-up period; patients with one complication was 9015 (48.10%) and 5888 (24.37%) during the same periods, respectively. Two or more complications were observed in 9726 (34.89%) and 18,269 (65.54%) patients during the same periods, respectively. Complications at the index date were the highest for neuropathy in 8896 (47.47%) followed by cardiovascular complications in 7508 (40.06%) and retinopathy in 5739 (30.62%). Patients with complications at the end of follow-up period were 24,157 of 27,871 (86.67%) with the highest for neuropathy in 15,385 (63.69%), followed by cardiovascular complications in 12,652 (52.37%) and retinopathy in 11,698 (48.42%) of 24,157 patients (Supplement figure 1, Supplement Table 3).

### Incidence and relative risk of new-onset diabetes complications during the follow-up period

New additional complications not present at baseline developed in 18,741 of 27,871 (67.14%) patients during the follow-up period with the highest for neuropathy 9913 (54.05%), followed by cardiovascular complications 7738 (42.19%) and retinopathy 7595 (41.41%) out of 18,741 patients (Table 2).

**Table 2:** New onset complications from 2 years after T2DM diagnosis to the end of follow up period

Number of complications	Number of patients, n (%)	Category of complications, n (%)						
		Nephropathy	Neuropathy	Retinopathy	Cerebrovascular disease	Cardiovascular disease	Peripheral vascular disease	Acute metabolic complications
1	7248 (39.52)	837 (4.56)	2204 (12.02)	1667 (9.09)	269 (1.47)	1410 (7.69)	823 (4.49)	38 (0.21)
2	5232 (28.53)	1195 (6.52)	2954 (16.11)	2120 (11.56)	633 (3.45)	2095 (11.42)	1411 (7.69)	56 (0.31)
3	3250 (17.72)	1203 (6.56)	2401 (13.09)	1804 (9.84)	814 (4.44)	2015 (10.99)	1450 (7.91)	63 (0.34)
4	1718 (4.00)	924 (5.04)	1509 (8.23)	1215 (6.63)	685 (2.46)	1372 (7.48)	1101 (6.00)	66 (0.36)
5	733 (2.63)	556 (3.0.)	688 (3.75)	632 (3.45)	448 (1.61)	688 (3.75)	609 (3.32)	44 (0.24)
6	150 (0.82)	149 (0.81)	149 (0.81)	149 (0.81)	140 (0.50)	150 (0.82)	146 (0.80)	17 (0.09)
7	8 (0.004)	8 (0.04)	8 (0.04)	8 (0.04)	8 (0.03)	8 (0.4	8 (0.04)	8 (0.04)
Total	18,339 (100)	4872 (26.57)	9913 (54.05)	7595 (41.41)	2997 (10.75)	7738 (42.19)	5548 (30.25)	292 (1.59)

The risk of new complications during the follow-up period increased with age, female, and a higher CCI score. As DCSI at index date increased, the risk of new complications increased by 26% [relative risk (RR) 1.26, 95% CI 1.25–1.27]. As expected the use of more than three antidiabetics was associated with the risk of new complications by 13% increase (RR 1.13, 95% CI 1.10–1.17); the risk was relatively higher in patients treated with insulin (RR 1.07, 95% CI 1.03–1.11) and relatively lower in patients treated with statins (RR 0.71, 95% CI 0.69–0.73) as the concomitant medication. On comparing several monotherapies of antidiabetic drugs with reference metformin, the risk of new complications was higher in patients treated with sulfonylurea (RR 1.29, 95% CI 1.27–1.32) than with DPP4-I (RR 0.77, 95% CI 0.70–0.85). On comparing dual therapy [metformin + SU, MS] with [metformin + DPP4-I, MD], patients treated with MD had a 29% (RR 0.71, 95% CI 0.69–0.73) lower risk of new complications (Table 3).

**Table 3:** Relative risk of new onset diabetic complications by patient characteristics

Characteristics	Composite complications RR (95% CI)
Age	1.006 (1.005–1.007)^a^
Men (Ref. women)	0.93 (0.92–0.95)^a^
CCI	1.018 (1.013–1.023)^a^
Baseline DCSI	1.26 (1.25–1.27)^a^
Insulin	1.07 (1.03–1.11)^a^
Aspirin	1.07 (1.06–1.09)^a^
Clopidogrel	0.96 (0.94–0.98)^a^
Sarpogrelate	1.00 (0.97–1.02)
Cilostazol	0.99 (0.95–1.03)
ACEI/ARB	0.99 (0.98–1.01)
Statin	0.92 (0.91–0.93)^a^
Number of antidiabetics	
1	Reference
2	1.01 (1.00–1.03)
3 +	1.13 (1.10–1.17)^a^
Antidiabetics therapy	
Mono therapy	
Metformin	Reference
DPP4-I	0.77 (0.70–0.85)^a^
Sulfonylurea	1.29 (1.27–1.32)^a^
Glucosidase	1.25 (1.18–1.33)^a^
Dual therapy	
Metformin + Sulfonylurea	Reference
Metformin + DPP4-I	0.71 (0.69–0.73)^a^

*Abbreviation*: *CCI* Charlson comorbidity index score, *ACEI/ARB* angiotensin-1 converting enzyme Inhibitor/angiotensin-2 receptor blocker, *DPP4-I* dipeptidyl peptidase-4 inhibitor

^a^ statistically significant

### All-cause hospitalization

A total of 14,297 (51.30%) of the 27,871 patients were admitted to the hospital with a frequency of 3.27 ± 5.0. The mean frequency of hospitalization during the follow-up period was 2.96 ± 5.69 in patients without complications, 3.15 ± 4.57 with one complication, and 3.59 ± 4.78 with two or more complications. The average duration of hospitalization was 54.94 days in patients without complications, 76.40 days with one complication, and 106 days with two or more complications (Supplement Table 4).

The adjusted RR of hospitalization was 1.06 (95% CI 1.01–1.11) when DCSI was 1, and it increased to 1.34 (95% CI 1.23–1.47) when DCSI was ≥5 compared with when DCSI was 0 as the reference (Table 4, Supplement Table 5).

**Table 4:** Relative risk of hospitalization and hazard ratio of mortality

Characteristics	Hospitalization adjusted RR^a^ (95%CI)	Mortality adjusted HR^a^ (95%CI)
Age	1.009 (1.008–1.011)	1.093 (1.088–1.097)
Men (Ref. women)	0.94 (0.90–0.97)	1.80 (1.65–1.96)
Insulin use	1.91 (1.81–2.02)	2.83 (2.52–3.17)
Diabetic complications severity index (linear)	1.04 (1.03–1.06)	1.13 (1.11–1.16)
DCSI (categorical)		
0	Reference	Reference
1	1.06 (1.01–1.11)	1.05 (0.93–1.19)
2	1.10 (1.04–1.16)	1.12 (0.98–1.27)
3	1.11 (1.03–1.18)	1.34 (1.17–1.54)
4	1.21 (1.12–1.32)	1.76 (1.51–2.05)
5+	1.34 (1.23–1.47)	1.87 (1.60–2.19)
Number of complications (categorical)		
0	Reference	Reference
1	1.06 (1.01–1.11)	1.10 (0.98–1.24)
2	1.10 (1.04–1.16)	1.29 (1.14–1.46)
3	1.16 (1.08–1.24)	1.45 (1.26–1.66)
4	1.22 (1.11–1.34)	1.57 (1.31–1.89)
5+	1.36 (1.18–1.57)	1.65 (1.24–2.20)

^a^Poisson regression model for relative risk (RR) hospitalization and Cox proportional hazard model for hazard ratio (HR) mortality, adjusted - age, sex, insulin use

### All-cause mortality

**Fig. 2:**
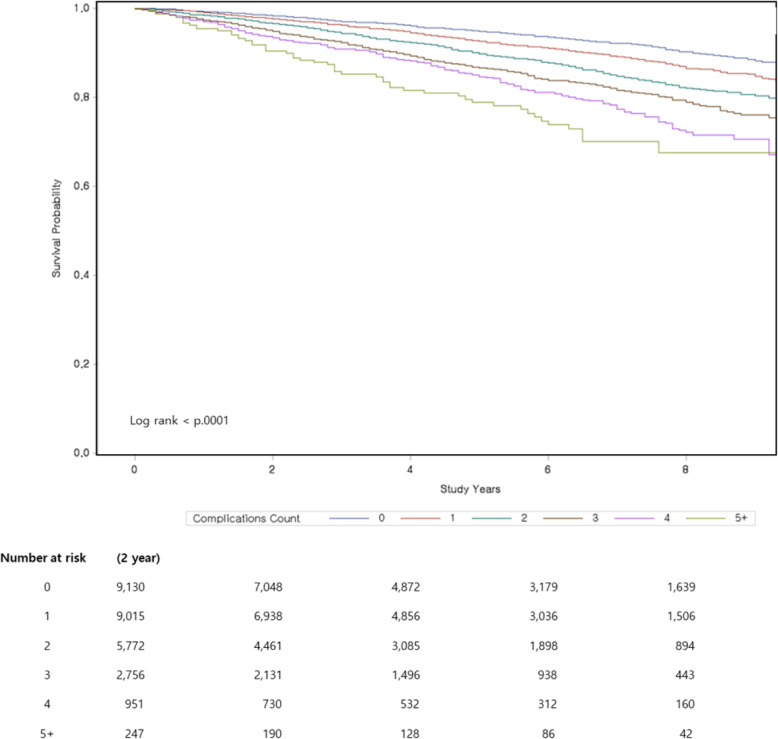
Kaplan-Meier survival curves stratified by number of complications for follow up period

Among the 27,871 patients, 2302 (8.26%) died because of all causes. Overall, 490 (5.37%) of the 9130 patients without complications, 659 (7.31%) of the 9015 patients with one complication, and 1153 (11.85%) of the 9726 patients with two or more complications died (Supplement Table 4). The ROC curve showed that DCSI was a useful indicator of mortality (Supplement figure 2). As the number of diabetes complications increased, the survival rate of patients decreased significantly (Fig. 2). The adjusted hazard ratio of mortality increased to 1.05 (95% CI 0.93–1.19) when DCSI was 1 and 1.87 (95% CI 1.60–2.19) when DCSI was ≥5 compared with when DCSI was 0 as the reference (Table 4, Supplement Table 6).

## Discussion

This study showed the clinical significance that DCSI including the number and severity of complications at 2 years after T2DM diagnosis was associated with mortality and hospitalization based on the real world data for a study period from 2002 to 2015. The hazard model and survival analysis using DCSI could be utilized for the stratification of risks for improving the strategy of medical care to prevent adverse events associated with DM complications. Patients with complications increased to 87% and the number and severity of complications increased as the duration of diabetes was longer during a mean follow-up period of 6.56 years. Approximately 50% of the complications occurred within 2 years of T2DM diagnosis (supplement figure 1). The higher the DCSI and number of complications were, the more the risk of mortality and hospitalization increased (Supplement Table 4–6) As the number of complications increases, the disease burden, mortality, and medical costs also increase [15, 17]. The management of diabetes complications as well as blood glucose control is crucial in diabetic patients [9, 12, 22]. Studies have reported on antidiabetic drugs, such as DPP4I and SGLT2I, which reduce or delay the occurrence of diabetes complications [23–28]. Furthermore, studies have evaluated the effects of cilostazol, sarpogrelate, and antiplatelets in preventing or delaying diabetic nephropathy [29, 30]. Statin has been recommended for primary and secondary prevention of cardiovascular and cerebrovascular disease with LDL-c levels below 100 mg / dL even in patients with type 2 diabetes [31]. The prevalence of dyslipidemia was 34.9% in patients with DM in the Korean population [32] and 72.6% in our study patients who had been strictly defined with T2DM diagnosis and with antidiabetic medications. They tried first with lifestyle modification followed by drug therapy. The use of statin therapy at the index date was 46.5% in our study patients which have been acceptable at the early stage of DM as a usual medical practice with health insurance reimbursement criteria in Korea. The risk of new DM complications was lower RR of 0.71 (95% CI, 0.69–0.73) in patients with statins in this study which implied to encourage statin use in the early stage within 2 years of T2DM diagnosis in Korea.

Young et al. analyzed diabetes complications in patients with DM. The patient group included both type 1 and type 2 DM and was analyzed for the risk of mortality until the end of the follow-up period of 4 years from 2001 to 2005. The risk of mortality did not increase significantly in patients with one complication but increased by 1.9 times in two complications, and 7.18 times in more than 5 complications. Similar results were obtained when DCSI was used instead of the number of diabetes complications [14]. Compared with a previous study, the present study used a sample cohort of the Korean National Health Insurance Claim database, analyzed the risk of mortality based on the number and severity of the complications at 2 years postdiagnosis in patients with new T2DM. The follow-up period after the index date of 2 years after the T2DM diagnosis was longer than that reported in a previous study. The duration of complications postdiagnosis was 2 years postdiagnosis for predicting the mortality in the patients, and the subsequent follow-up period ranged from a minimum of 2 years to a maximum of 12 years. To minimize the effects of death due to reasons other than diabetes complications, patients with cancer, severe liver failure, and severe renal dysfunction requiring dialysis or kidney transplantation were excluded from the study. Although the previous and the present studies were different in terms of the inclusion criteria, exclusion criteria, and follow up period, it was confirmed that the risk of mortality increased significantly as the number and severity of complications increased compared with the patients with no or fewer diabetes complications.

The present study used the National Health Insurance Claim database which is based on the available claim and demographics data held by the National Health Insurance Corporation (NHIC) and is representative of the Korean population. This study was a retrospective cohort design, which reflected the process of diabetes diagnosis and treatment in the real world clinical practice. The study design included a postdiagnosis period of 2 years as the run-in period before the index date. The complications at 2-year postdiagnosis as the index date were the baseline clinical information associated with the mortality and hospitalization during the follow-up period. The present study also analyzed the factors associated with the complications, which included comorbidity, type of antidiabetic drugs, the pattern of pharmacotherapy, and concomitant medications. The risk of hospitalization or mortality was assessed by levels of zero to 5 or more DCSI, which reflects the number and severity of diabetes complications. Diabetes patients can be stratified according to their severity of morbidity through indicators such as DCSI with HbA1C or blood glucose levels. The utilization of DCSI in assessing patients with DM can improve the strategy in medical care and medication therapy management with the intensified consultation and proper reimbursement for the services. In the case of chronic diseases such as diabetes, medical benefits or services need to be differentiated according to the severity of complications. More medical insurance benefits could be provided for patients with severe diseases, which would save the direct or indirect medical costs. Clinical excellence in the medical care of DM patients would use efficiently the medical resources. The National Health Insurance System (NHIS) is operated by a single insurer for the entire nation with universal insurance and a mandatory service as a social security system in Korea. The public pays health insurance premiums to the NHIC by differentiating them according to income. When you see a doctor in a hospital or take a prescription drug from a pharmacy, you pay 30% as a copayment and a medical institution receives a 70% national contribution of the medical bill claims.

The present study had some limitations. The NHID data comprises health insurance claim data as well as the 2-year regular health examination data. This study could not include clinical laboratory tests related to the complications. The DM complications could not be analyzed on the basis of the clinical test results for glycosylated hemoglobin, blood glucose, blood pressure, lipid, and glomerular filtration rate because the NHID is not an electronic medical record of hospital patients. The health examination data included in the NHID is the result of regular check-ups every 2 years, independent of medical care in a hospital. However, the concurrent medications and diseases were included in the analyses instead of the laboratory tests to evaluate the effects of the clinical factors on the incidence of complications and the risk of mortality or hospitalization. Besides, we could not use the information on weight, diet, genetic factors, smoking status, and vaccination, which were the risk factors for diabetes complications. In the present study, the occurrence of complications was identified using only diagnostic codes comprising 55 subcategories of 7 categories, which reflected the diabetes complications evaluated in a previous study [14] as much as possible. The complications were measured at 2 years after T2DM diagnosis to match with the duration of diabetes among the study subjects with a relatively longer follow-up out of the study period. The baseline complications at 5 or 10 years after T2DM diagnosis would also be interesting for evaluating the risks of mortality and hospitalization for a shorter follow-up period.

## Conclusions

In this retrospective cohort study using the claim data of the National Health Insurance Sample Cohort as the real world data, DCSI including the number and severity of DM complications showed the association of complications with mortality and hospitalization in patients with diabetes. The incidence of complications was different based on coexisting diseases, hypoglycemic agents, and concomitant medications. DCSI can be utilized to assess and care appropriately for patients with DM in addition to hemoglobin A1c and blood glucose level.

## Supplementary information

**Additional file 1 : Supplement Table 1.** Diabetes complications severity index and list of complications developed from KCD-7 code. **Supplement Table 2.** Drug use patterns in patients without and with new onset complications from 2 years after DM diagnosis to the end of follow up period. **Supplement Table 3.** Number and category of complications at 2 years after T2DM diagnosis and the end of the follow-up period. **Supplement Table 4.** All-cause mortality and hospitalization. **Supplement Table 5.** Relative risk of hospitalization with Poisson regression model. **Supplement Table 6.** Hazard ratio of mortality with Cox proportional hazard model. **Supplement figure 1.** Changes in category of diabetic complications according to duration of diabetes mellitus (A), Changes in number of diabetic complications according to duration of diabetes mellitus (B). **Supplement figure 2.** Receiver operating characteristics (ROC) curves comparing the diabetic complications severity index (DCSI) with complications count (blue). The lines of DCSI (dotted red) and DCSI linear (dotted green) were overlapped.

## Data Availability

The population-based cohort data that support the findings of this study are available from the Korean National Health Insurance Service (NHIS) but restrictions apply to the availability of these data, which were used under license for the current study with online access to the Big Data Center server of NHIS through a designated computer and so are not publicly available. Only results of analysis only could be taken out and analysis could be repeated once for validation on request by authors.

## Abbreviations

T2DM: Type 2 diabetes mellitus
NHIS-NSC: National Health Insurance Service–National Sample Cohort
NHID: National Health Insurance Database
DCSI: Diabetes complications severity index
SU: Sulfonylurea
DPP4I: Dipeptidyl peptidase-4 inhibitor
ACEI/ARB: Angiotensin-1 converting enzyme inhibitor/angiotensin-2 receptor blocker
DHP-CCB: Dihydropyridine-calcium channel blocker
