# Supplementary material for: Study of hospitalization and mortality in Korean diabetic patients using the diabetes complications severity index
Source: BMC Endocr Disord. 2020 Aug 10;20:122. doi: 10.1186/s12902-020-00605-5 (PMC7418200; doi:10.1186/s12902-020-00605-5)
Supplement: Supplementary file 1 — Additional file 1 : Supplement Table 1. Diabetes complications severity index and list of complications developed from KCD-7 code. Supplement Table 2. Drug use patterns in patients without and with new onset complications from 2 years after DM diagnosis to the end of follow up period. Supplement Table 3. Number and category of complications at 2 years after T2DM diagnosis and the end of the follow-up period. Supplement Table 4. All-cause mortality and hospitalization. Supplement Table 5. Relative risk of hospitalization with Poisson regression model. Supplement Table 6. Hazard ratio of mortality with Cox proportional hazard model. Supplement figure 1. Changes in category of diabetic complications according to duration of diabetes mellitus (A), Changes in number of diabetic complications according to duration of diabetes mellitus (B). Supplement figure 2. Receiver operating characteristics (ROC) curves comparing the diabetic complications severity index (DCSI) with complications count (blue). The lines of DCSI (dotted red) and DCSI linear (dotted green) were overlapped. [file 12902_2020_605_MOESM1_ESM.docx]

Supplement Table 1. Diabetes complications severity index and list of complications developed from KCD-7 code

| Category | Code | Description | KCD-7 | DCSI score |
| --- | --- | --- | --- | --- |
| Nephropathy | 11 | Diabetic nephropathy | E112, H1020, H1121, N083 | 1 |
|  | 12 | Acute glomerulonephritis | N008, N009, N019, | 1 |
|  | 13 | Chronic glomerulonephritis | N038, N031, N035, N039 | 1 |
|  | 14 | Glomerular disorders in other disease classified | N088 | 1 |
|  | 15 | Nephrotic syndrome | N040, N042, N045, N048, N049 | 1 |
|  | 16 | Nephritis/nephropathy | N052, N055, N058, N059 | 1 |
|  | 17 | Chronic renal failure | N189 | 2 |
|  | 18 | Unspecified kidney failure | N19 | 2 |
|  | 19 | Renal insufficiency | N289 | 2 |
| Neuropathy | 21 | Diabetic neuropathy | E114, G609 | 1 |
|  | 22 | Cranial nerve palsy | S041, S042, S044 | 1 |
|  | 23 | Mono-neuropathy | G56, G57, G58 | 1 |
|  | 24 | Polyneuropathy | G632 | 1 |
|  | 25 | Neuropathic arthropathy | M146 | 1 |
|  | 26 | Neurogenic bladder | N319 | 1 |
|  | 27 | Autonomic neuropathy | K591, K3188 | 1 |
|  | 28 | Orthostatic hypotension | I951 | 1 |
| Retinopathy | 31 | Diabetic ophthalmologic disease | E113 | 1 |
|  | 32 | Background retinopathy | H350 | 1 |
|  | 33 | Other retinopathy | H360 | 1 |
|  | 34 | Retinal edema | H358 | 1 |
|  | 35 | CSME (clinically significant macular edema) | H353 | 1 |
|  | 36 | Other retinal disorders | H356 | 1 |
|  | 37 | Vitreous hemorrhage | H431 | 1 |
|  | 38 | Retinal detachment | H33 | 2 |
|  | 39 | Blindness | H540 | 2 |
| Cerebrovascular disease | 41 | Transient cerebral ischemic attacks | G45 | 1 |
|  | 42 | Stroke | I61, I63, I64 | 2 |
| Cardiovascular disease | 50 | Atherosclerosis | I70 | 1 |
|  | 51 | Angina pectoris | I20 | 1 |
|  | 52 | Other acute ischemic heart diseases | I24 | 1 |
|  | 53 | Chronic ischemic heart diseases | I25 | 1 |
|  | 54 | Other aneurysm, lower extreme | I72 | 1 |
|  | 55 | Myocardial infarction | I21 | 2 |
|  | 56 | Ventricular fibrillation, arrest | I472 | 2 |
|  | 57 | Atrial fibrillation, arrest | I48, I49, I46 | 2 |
|  | 58 | Congestive heart failure | I500 | 2 |
|  | 59 | Aortic aneurysm, dissection | I71 | 2 |
| Peripheral vascular disease | 61 | Diabetic PVD | E1151, I792 | 1 |
|  | 62 | PVD | I739 | 1 |
|  | 63 | Embolism/thrombosis (LE) | I743 | 2 |
|  | 64 | Gangrene | R02 | 2 |
|  | 65 | Ulcer of lower limbs | L97 | 2 |
| Acute metabolic complications | 71 | Ketoacidosis | E1110 | 2 |
|  | 72 | Hyperosmolar | E1100 | 2 |
|  | 73 | Other coma | E1101, E1102, E1103, E1104 | 2 |

Supplement Table 2. Drug use patterns in patients without and with new onset complications from 2 years after DM diagnosis to the end of follow up period

| Medications, n (%) | All (n=27,871) | New onset complications | |
| --- | --- | --- | --- |
|  |  | Without (n=9,532) | With  (n=18,339) |
| Antidiabetics class |  |  |  |
| Metformin | 19,526 (70.06) | 7,307 (76.66) | 12,219 (66.63) |
| Sulfonylurea | 14,945 (53.62) | 3,854 (40.43) | 11,091 (60.48) |
| DPP4-I | 4,314 (15.48) | 2,421 (25.40) | 1,893 (10.32) |
| α-glucosidase inhibitor | 2,416 (8.67) | 438 (4.60) | 1,978 (10.79) |
| Thiazolidinedione | 1,703 (6.11) | 466 (4.89) | 1,237 (6.75) |
| Insulin | 715 (2.57) | 159 (1.67) | 446 (4.59) |
| Number of antidiabetics |  |  |  |
| 1 | 1,2512 (44.89) | 4,122 (43.24) | 8,390 (45.75) |
| 2 | 11,726 (42.07) | 4,088 (42.89) | 7,638 (41.65) |
| 3 + | 1,701 (6.10) | 532 (5.58) | 1,169 (6.37) |
| Others^*^ | 1,932 (6.93) | 790 (8.29) | 1,142 (6.23) |
| Antidiabetics therapy |  |  |  |
| Monotherapy |  |  |  |
| Metformin | 6,693 (24.01) | 2,825 (29.64) | 3,868 (29.64) |
| Sulfonylurea | 5,017 (18.00) | 1,025 (10.75) | 3,992 (10.75) |
| α-glucosidase inhibitor | 353 (1.27) | 76 (0.80) | 277 (1.51) |
| DPP4-I | 212 (0.76) | 144 (1.51) | 68 (0.37) |
| Dual therapy |  |  |  |
| Metformin + Sulfonylurea | 6,450 (23.14) | 1,866 (19.57) | 6,450 (25.00) |
| Metformin + DPP4-I | 12,512 (10.81) | 1,689 (17.72) | 1,325 (7.23) |
| Cardiovascular agents |  |  |  |
| Aspirin | 8,791 (31.54) | 2,543 (26.68) | 6,248 (34.07) |
| Clopidogrel | 1,754 (6.29) | 567 (5.95) | 1,187 (6.47) |
| Cilostazol | 1,068 (3.83) | 203 (2.13) | 865 (4.72) |
| Sarpogrelate | 726 (2.60) | 195 (2.05) | 531 (2.90) |
| ACEI/ARB | 13,325 (47.81) | 4,484 (47.04) | 8,841 (48.21) |
| DHPCCB | 11,336 (40.67) | 3,545 (37.19) | 7,791 (42.48) |
| Statin | 12,966 (46.52) | 4,682 (49.12) | 8,284 (45.17) |
| Per oral drug use history is prescribed more than 90 days before one year at 2 years after new onset type 2 DM.  * others: diabetes drug prescription is less than 90 days  Abbreviation: DPP4-I, Dipeptidyl peptidase-4 inhibitor; ACEI/ARB, angiotensin-1 converting enzyme Inhibitor/angiotensin-2 receptor blocker; DHPCCB, dihydropyridine-calcium channel blocker | | | |

Supplement Table 3. Number and category of complications at 2 years after T2DM diagnosis and the end of the follow-up period

| Patients | 2 years after DM diagnosis (n=27,871) | | | | | | | | End of follow-up period (n=27,871) | | | | | | | | |  |
| --- | --- | --- | --- | --- | --- | --- | --- | --- | --- | --- | --- | --- | --- | --- | --- | --- | --- | --- |
| Without complications n (%) | 9,130 (32.76) | | | | | | | | 3,714 (13.33) | | | | | | | | |  |
| With complications  n (%) | 18,741 (67.24) | | | | | | | | 24,157 (86.67) | | | | | | | | |  |
|  | Category of complications, n (%) | | | | | | | | | | | | | | | | | |
| Number of complications | Patients  n (%) | I | II | III | IV | V | VI | VII | | Patients  n (%) | I | II | III | IV | V | VI | VII | |
| 1 | 9,015  (48.10) | 989  (5.28) | 2,731  (14.57) | 1,620  (8.64) | 572  (3.05) | 2,044  (10.91) | 1,031  (5.50) | 28  (0.15) | | 5,888  (24.37) | 738  (3.06) | 1,798  (7.44) | 1,270  (5.26) | 257  (1.06) | 1,127  (4.67) | 668  (2.77) | 30  (0.12) | |
| 2 | 5,772  (30.80) | 1,314  (7.01) | 3,061  (16.33) | 1,963  (10.47) | 975  (5.20) | 2,655  (14.17) | 1,537  (8.20) | 39  (0.21) | | 6,404  (26.51) | 1,534  (6.35) | 3,563  (14.75) | 2,530  (10.47) | 779  (3.22) | 2,591  (10.73) | 1,738  (7.19) | 73  (0.30) | |
| 3 | 2,756  (14.71) | 995  (5.31) | 2,036  (10.86) | 1,322  (7.05) | 899  (4.80) | 1,792  (9.56) | 1,200  (6.40) | 24  (0.13) | | 5,491  (22.73) | 1,980  (8.20) | 4,145  (17.16) | 2,938  (12.16) | 1,416  (5.86) | 3,388  (14.02) | 2,538  (10.51) | 68  (0.28) | |
| 4 | 951  (5.07) | 511  (2.73) | 828  (4.42) | 630  (3.36) | 484  (2.58) | 778  (4.15) | 553  (2.95) | 20  (0.11) | | 3,766  (15.59) | 1,930  (7.99) | 3,342  (13.83) | 2,630  (10.89) | 1,540  (6.37) | 3,065  (12.69) | 2,463  (10.20) | 94  (0.39) | |
| 5 | 211  (1.13) | 151  (0.81) | 204  (1.09) | 168  (0.90) | 162  (0.86) | 203  (1.08) | 161  (0.86) | 6  (0.03) | | 1,938  (8.02) | 1,446  (5.99) | 1,867  (7.73) | 1,663  (6.88) | 1,194  (4.94) | 1,812  (7.50) | 1,629  (6.50) | 79  (0.33) | |
| 6 | 35  (0.19) | 35  (0.19) | 35  (0.19) | 35  (0.19) | 35  (0.19) | 35  (0.19) | 35  (0.19) | 0  (0.00) | | 651  (2.69) | 642  (2.66) | 651  (2.69) | 648  (2.68) | 625  (2.59) | 650  (2.69) | 643  (2.69) | 47  (0.19) | |
| 7 | 1  (0.01) | 1  (0.01) | 1  (0.01) | 1  (0.01) | 1  (0.01) | 1  (0.01) | 1  (0.01) | 1  (0.01) | | 19  (0.08) | 19  (0.08) | 19  (0.08) | 19  (0.08) | 19  (0.08) | 19  (0.08) | 19  (0.08) | 19  (0.08) | |
| Total | 18,741  (100) | 3,996  (21.32) | 8,896  (47.47) | 5,739  (30.62) | 3,128  (16.69) | 7,508  (40.06) | 4,518  (24.11) | 118  (0.63) | | 24,157  (100) | 8,289  (34.31) | 15,385  (63.69) | 11,698  (48.42) | 5,830  (24.13) | 12,652  (52.37) | 9,698  (40.15) | 410  (1.70) | |
| Diabetic complication category  I : nephropathy, II : neuropathy, III : retinopathy, IV : cerebrovascular disease, V : cardiovascular disease, VI : peripheral vascular disease, VII : acute metabolic complications | | | | | | | | | | | | | | | | | | |

Supplement Table 4. All-cause mortality and hospitalization

| Characters | All subjects  (27,871) | Patients with complications at 2 years of diagnosis | | |
| --- | --- | --- | --- | --- |
|  |  | No (9,130) | 1 (9,015) | ≥ 2 (9,726) |
| All cause death  n, (%) | 2,302 (8.26) | 490 (5.37) | 659 (7.31) | 1,153 (11.85) |
| All cause hospitalization  n, (%) | 14,297 (51.30) | 3,977 (43.56) | 4,491 (49.82) | 5,829 (59.93) |
| Frequency,  Mean ±SD  Median, IQR | 3.27±5.0  2, 1-4 | 2.96±5.69  2, 1-3 | 3.15±4.57  2, 1-4 | 3.59±4.78  2, 1-4 |
| Period (days)  Mean ±SD  Median, IQR | 82.86±248.97  16, 5-49 | 54.94±180.84  12, 4-33 | 76.40±242.62  15, 5-45 | 106.89±288.54  20, 6-66 |
| Results were not normal distribution (Normality test - Kolmogorov-Smirnov) | | | | |

Supplement Table 5. Relative risk of hospitalization with Poisson regression model

| Characteristics | Unadjusted RR (95%CI) | Adjusted RR (95%CI) |
| --- | --- | --- |
| Age | 1.015 (1.013-1.016) | 1.009 (1.008-1.011) |
| Men (Ref. women) | 0.84 (0.81-0.87) | 0.94 (0.90-0.97) |
| Insulin use | 2.31 (2.18-2.43) | 1.91 (1.81-2.02) |
| Diabetic complications severity index (linear) | 1.17 (1.16-1.18) | 1.04 (1.03-1.06) |
| DCSI (categorical) |  |  |
| 0 | Reference | Reference |
| 1 | 1.19 (1.14-1.33) | 1.06 (1.01-1.11) |
| 2 | 1.38 (1.31-1.46) | 1.10 (1.04-1.16) |
| 3 | 1.55 (1.46-1.65) | 1.11 (1.03-1.18) |
| 4 | 1.92 (1.79-2.06) | 1.21 (1.12-1.32) |
| 5+ | 2.60 (2.42-2.80) | 1.34 (1.23-1.47) |
| Number of Complications (categorical) |  |  |
| 0 | Reference | Reference |
| 1 | 1.22 (1.16-1.28) | 1.06 (1.01-1.11) |
| 2 | 1.47 (1.39-1.54) | 1.10 (1.04-1.16) |
| 3 | 1.78 (1.67-1.89) | 1.16 (1.08-1.24) |
| 4 | 2.21 (2.04-2.40) | 1.22 (1.11-1.34) |
| 5+ | 3.05 (2.68-3.47) | 1.36 (1.18-1.57) |
| adjusted - age, sex, insulin use | | |

Supplement Table 6. Hazard ratio of mortality with Cox proportional hazard model

| Characteristics | Unadjusted HR (95%CI) | Adjusted HR (95%CI) |
| --- | --- | --- |
| Age | 1.088 (1.084-1.093) | 1.093 (1.088-1.097) |
| Men (Ref. women) | 0.99 (0.91-1.07) | 1.80 (1.65-1.96) |
| Insulin use | 3.12 (2.80-3.49) | 2.83 (2.52-3.17) |
| Diabetic complications severity index (linear) | 1.32 (1.28-1.35) | 1.13 (1.11-1.16) |
| DCSI (categorical) |  |  |
| 0 | Reference | Reference |
| 1 | 1.23 (1.09-1.40) | 1.05 (0.93-1.19) |
| 2 | 1.58 (1.39-1.80) | 1.12 (0.98-1.27) |
| 3 | 2.16 (1.89-2.48) | 1.34 (1.17-1.54) |
| 4 | 3.33 (2.86-3.86) | 1.76 (1.51-2.05) |
| 5+ | 4.39 (3.78-5.10) | 1.87 (1.60-2.19) |
| Number of complications (categorical) |  |  |
| 0 | Reference | Reference |
| 1 | 1.38 (1.23-1.56) | 1.10 (0.98-1.24) |
| 2 | 1.92 (1.70-2.16) | 1.29 (1.14-1.46) |
| 3 | 2.50 (2.18-2.86) | 1.45 (1.26-1.66) |
| 4 | 3.15 (2.63-3.76) | 1.57 (1.31-1.89) |
| 5+ | 4.16 (3.14-5.51) | 1.65 (1.24-2.20) |
| adjusted - age, sex, insulin use |  |  |

**A** **B**

Supplement figure 1. Changes in category of diabetic complications according to duration of diabetes mellitus (A), Changes in number of diabetic complications according to duration of diabetes mellitus (B)


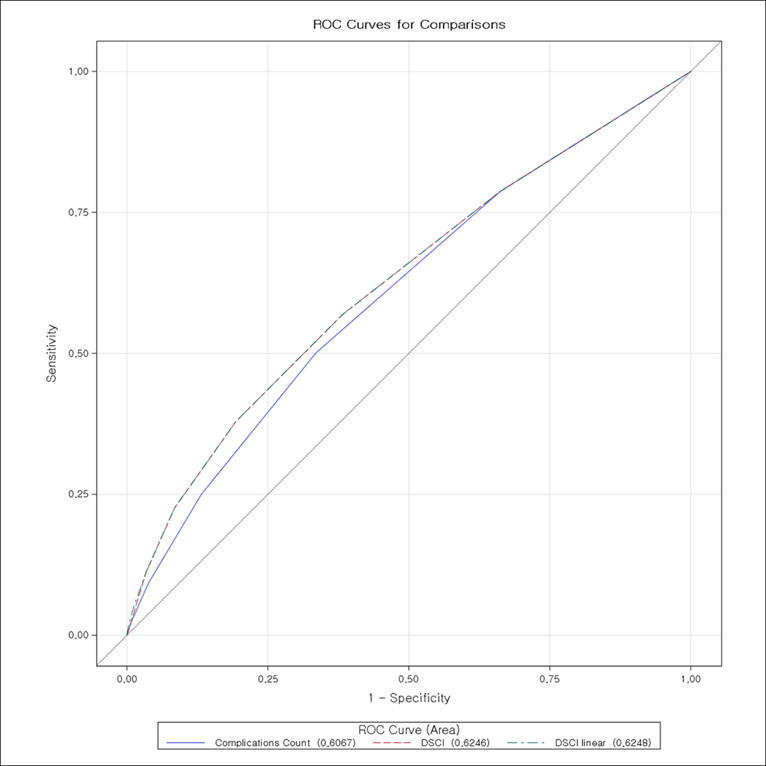


| ROC contrast test results | p-value |
| --- | --- |
| Complications count : reference | < .0001 |
| DCSI : complications count | < .0001 |
| DCSI linear : complications count | < .0001 |

Supplement figure 2. Receiver operating characteristics (ROC) curves comparing the diabetic complications severity index (DCSI) with complications count (blue). The lines of DCSI (dotted red)and DCSI linear (dotted green) were overlapped.
